# Phishing website prediction using base and ensemble classifier techniques with cross-validation

**DOI:** 10.1186/s42400-022-00126-9

**Published:** 2022-11-02

**Authors:** Anjaneya Awasthi, Noopur Goel

**Affiliations:** Department of Computer Applications, VBS Purvanchal University, Jaunpur, UP India

**Keywords:** Phishing, Hacking, Data diddling, Machine learning, Ensemble

## Abstract

Internet or public internetwork has become a vulnerable place nowadays as there are so many threats available for the novice or careless users because there exist many types of tools and techniques being used by notorious people on it to victimize people somehow and gain access to their precious and personal data resulting in sometimes smaller. However, these victims suffer considerable losses in many instances due to their entrapment in such traps as hacking, cracking, data diddling, Trojan attacks, web jacking, salami attacks, and phishing. Therefore, despite the web users and the software and application developer's continuous effort to make and keep the IT infrastructure safe and secure using many techniques, including encryption, digital signatures, digital certificates, etc. this paper focuses on the problem of phishing to detect and predict phishing websites URLs, primary machine learning classifiers and new ensemble-based techniques are used on 2 distinct datasets. Again on a merged dataset, this study is conducted in 3 phases. First, they include classification using base classifiers, Ensemble classifiers, and then ensemble classifiers are tested with and without cross-validation. Finally, their performance is analyzed, and the results are presented at last to help others use this study for their upcoming research.

## Introduction

The use of the internet is proliferating in our lives, and we are becoming so very dependent on the services provided online. From online shopping banking to intelligent home solutions, the working culture of people has also been affected, and as a result, the number of threats is also growing at a comparable pace. There exist so many kinds of threats on these globally operated network platforms. Apart from the well-known terms like hacking, cracking, web jacking, online terrorist organizations, one of the prevalent threats is phishing. Phishing is a way of committing crime online, but unfortunately, the victims of such attacks are either unaware of these attacks or do not pay ample attention to them. Such attacks target two types of users, first who are newbies that, means, they are not aware of the underlying technical aspects of the internet, and the others are those who are careless enough so they may understand the associated risks, but as they are careless, they don't even pay attention.

As per the 2020 Phishing Attack Landscape Report from Great horn (2020 Phishing Attack Landscape 2020), about 53 percent of cyber security professionals have stated that they have witnessed a spike in these attacks during COVID 19 Pandemic, and enterprises are facing about 1185 phishing attacks every month. It takes enterprise security teams to spend 1–4 days remediation a cyber-attack. According to the same report, about 30 percent of cyber security experts, phishing attacks gained tremendous success during this pandemic (2020 Phishing Attack Landscape 2020). Their study revealed the number of phishing emails targeting organizations worldwide (2020 Phishing Attack Landscape 2020). A large portion of phishing attacks yield success, and the productivity and profit also declined due to the time spent on remediation of such attacks. So in light of these facts, it is suggested that companies must educate their employees about the phishing threat.

The study in this paper is a try for providing the possible solution for the prediction of phishing websites, using URL features that may eventually be useful for further study. This paper is organized into 6 sections, where "Introduction" section contains an introduction and "Motivation" section comprises Motivation. "Literature review" section includes the Literature review of previous research papers and studies conducted by other researchers worldwide. "Methodology" section comprises of Methodology that has been adopted in this paper. "Results and discussion" section includes results and a discussion showing all the results and outcomes of this study. Finally, "Conclusion and future scope" section comprises the results. The machine learning approaches and their outcomes are detailed in this paper as well as the characteristics and other details about individual algorithms are also provided.

## Motivation

Phishing is one of the most dangerous and hideous kinds of malicious acts performed in the internet world. However, many other internetwork security threats exist in today's internet workplace. Internet is referred to as a workplace because of our ever-growing reliance on data, networks, and related technologies. Many analysts and researchers are working rigorously may or may not be associated with an organization related to phishing attacks research. However, the objective of all of these people and organizations is the same, i.e., fighting against the phishing threat. Most of the time, the activities performed by the notorious cybercriminals prove to be successful in the absence of a proven mechanism that may give the people the rightly predicted information at the right time or as and when required. Machine learning-based research and models can play a vital role in developing such tools.

The phishing website prediction becomes part of the researcher's discussion. The study of Gupta et al. (Gupta et al. 2021) works on phishing website prediction. BB Gupta et al. uses 9 features with 4 classifier algorithms, i.e., Random Forest, KNN, SVM, and Logistic Regression.

The study of Sahingoz et al. (2019) uses7 algorithms, i.e., Decision Tree, Adaboost, Kstar, KNN, Random forest, SMO, and Naïve Bayes, to predict phishing websites using word Vector, hybrid, and NLP based features.

The study conducted by Jain and Gupta (2018a) uses 19 features 5 classifiers i.e. Random Forest, SVM, Neural Networks, Logistic Regression, Naïve Bayes for phishing website prediction using URL-based features.

The study of Moghimi et al. (2016) proposes a browser add-on extension for detecting phishing websites that uses a rule-based approach for URL features and an SVM classifier.

The presented research paper studies 12 machine learning classifier algorithms classified into 2 classes; ensemble and base classifiers. All 12 machine learning classifier algorithms use all 30 features to find the best predicting algorithm for predicting phishing websites.

## Literature review

Different authors have conducted many studies, and many researchers have done many pieces of research to detect and predict phishing websites based on different approaches at different times. For example, some of the researchers have come up with visual features, while some have suggested an image-based approach, logos are also considered to be one of the bases for detection, some of the researchers suggested HTML based features to be examined for this purpose while some of them have also suggested blacklisting and whitelisting of domains. The present paper proposes a URL feature-based approach to get these websites detected and predicted as if they are phishing websites or non-phishing ones. The URL dataset is taken from the UCI machine learning repository (2020). And the second dataset has been taken from Kaggle Repository (Phishing website dataset | Kaggle 2020). This section discusses previous research conducted by researchers and their detailed description of the work and their tools.

Hong et al. (2020) introduced an approach to perform phishing URL detection using lexical features and blacklist domains using Adaboost, Random Forest, and SVM. They have also used 3 string-based classifiers; and deep learning classifiers: 1DConv, LSTM, and 1DConv + LSTM.

Orunsolu et al. (2020) have proposed a feature selection-based approach using Support Vector Machines and Naïve Bayes machine learning classifiers using the weka tool to predict phishing URLs.

Aassal et al. (2020) have proposed a benchmarking and evaluation of phishing detection research studies, and the model was named PhishBench. In the proposed approach, they used term frequency-inverse document frequency (TF-IDF) (Shiri 2004), a well-known statistical tool that is a term weighting scheme that uses term frequency in a document and log value of inverse popularity. Their study also integrated 2 automated machine learning (AutoML) libraries, namely AutoSklearn, tree-based pipeline optimization tool (TPOT).

Sonowal and Kuppusamy (2020) have come up with their developed model named PhiDMA. It is a multi-filter model comprised of five layers—A whitelist layer, features layer, lexical signature, string matching, and a comparison layer for accessibility score. They also introduced their algorithm to predict phishing website URLs.

Abutair et al. (2019) introduced a case-based reasoning approach for phishing detection and named it CBR-PDS. They have used feature extraction and blacklisting of URLs and a genetic algorithm weighing technique to predict the phishing URLs.

Satapathy et al. (2019) proposed phishing detection model based on feature classification using machine learning and also used artificial neural network (ANN) along with Naïve Bayes, extreme learning machines (ELM).

Chin et al. (2018) presented their approach, Phishlimiter, phishing detection, and mitigation approach developed using software defined networking (SDN) and global environment for network innovation (GENI), a tested testbed environment. They have also tested support vector machines (SVM), J48 trees, Naïve–Bayes, and Logistic regression algorithms making up ANN environment for phishing detection.

Jain and Gupta (2018b) have proposed their work and named it PHISH-SAFE, a feature-based phishing URLs detection system using Machine learning techniques. They trained their model using support vector machines (SVM) and Naïve Bayes classifiers.

Kumar and Gupta (2018) also discussed an approach based on hyperlinks information to detect phishing website URLs. They have used feature selection and CSS and various machine learning classification algorithms such as SMO, Naïve Bayes, Random Forest, support vector machine (SVM), Adaboost, Neural Networks, C4.5, and Logistic Regression on WEKA tool to predict the phishing website URLs.

Gupta and Singhal (2018) have discussed their approach for detecting phishing URLs from a dataset taken from UCI online repository, and they employed at least 5 machine learning classifiers for their experiment. They used Random Tree, J48, Random Forest, Naïve Bayes, and LMT.

Abdelhamid and Abdel-jaber (2017) have discussed a phishing detection approach based on the model's content and features. To perform their experimental study, they used various machine learning classifiers, namely C4.5, OneRule, Conjunctive Rule, eDRI, RIDOR, Bayes Net, SVM, and Boosting.

Shirazi et al. (2017) have introduced their framework named Fresh-Phish for auto-detection of phishing websites. They experimented on Whois data taken from whoixmlsapi.com (WHOIS API gives access to domain registration records | WhoisXML API 2020) containing 6000 website's data and implemented 4 classifiers Tfcontrib (2020) library, and 2 classifiers from the sci-kit-learn library (Varoquaux et al. 2015) for comparison of accuracy. Further, they have also used TensorFlow and TFcontrib to build a deep neural network (DNN) with built-in optimizations, such as Adadelta, Adagrad, and Gradient Descent; lastly, they used support vector machines (SVM) with stratified K fold for validation and grid search to get the phishing website URLs predicted for their phishing or non-phishing nature.

Leng et al. (2019) introduced their ensemble-based feature selection framework implemented with a machine learning approach. They used the WEKA tool with ML algorithms such as SVM, Random Forest, Naïve Bayes, etc., along with Cumulative Distribution function for feature selection and classified with the algorithms as above. The web URL data was taken from phishtank.com (PhishTank | Join the fight against phishing 2020).

Srinivasa et al. (2019) presented a two-level filtering mechanism for detecting phishing websites by first getting the domain information extracted then performing feature selection. Next, they applied hash functions on each one of them. Then they compared the data with blacklisted URLs. If a match is found, then announce it as a phishing website, and if it doesn't, they applied heuristics after that with machine learning classification algorithms to achieve the comparison.

Mao (2019) discussed a Phishing detection model using page layout features and classifiers. The dataset contains 490 phishing websites is taken from Phishtank.com, using 4 Machine Learning classifiers, namely support vector machine (SVM), decision tree (DT), random forest (RFC), and AdaBoost; CSS is used for page layout, and classifier's training is performed on vector-based data.

Ali (2017) have introduced an approach based on supervised machine learning algorithms for phishing website detection. This study included the wrapper feature selection with the help of machine learning classifiers such as Back-propagation neural network (BPNN), radial basis function network (RBFN), Naïve Bayes (NB) support vector machines (SVM), Decision Tree (C4.5), K-nearest neighbor (k-NN) and random forest (RF). Using knowledge and content information gained from this literature review, the present paper and its experiments have been carried out.

## Methodology

In the present paper, there are 3 experiments conducted, and their performance is displayed in the "Results and discussion" section of this paper.

**Fig. 1:**
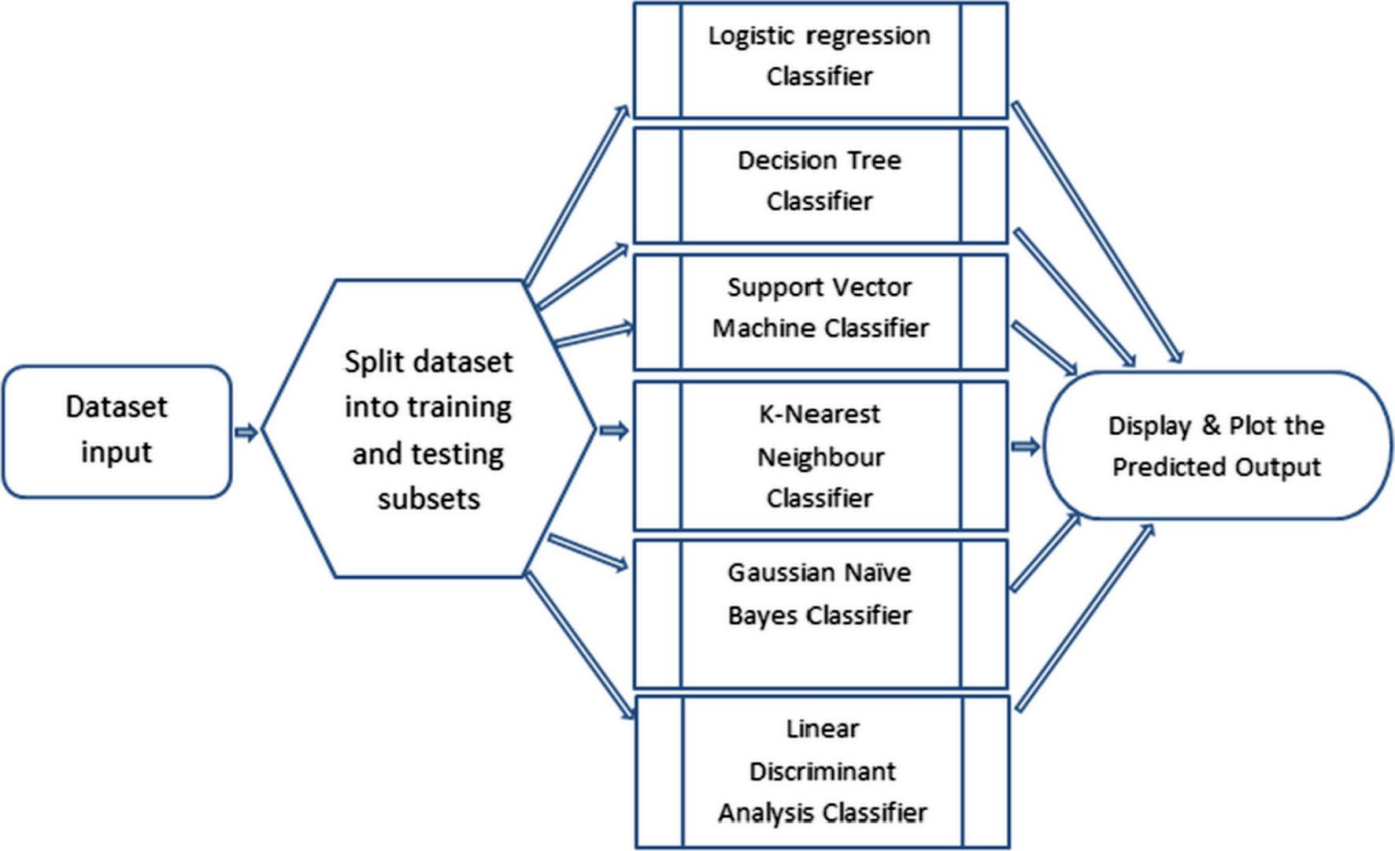
Experiment 1 Base classifiers process chart

*The Base Classifiers* The base machine learning classifiers used in this experiment are: at first the logistic regression classifier is used, second the Gaussian Naïve Bayes classifier, next the Decision Tree Classifier is used, next Support Vector Machine classifier, and next to The K-Nearest Neighbor classifier next Linear Discriminant Analysis classifiers are used. The process chart of these base classifiers is shown below in Fig. 1. All the individual base classifiers are described next to the process chart.

### Base Classifiers used

1. Logistic regression Classifier

The logistic function was earlier named "Logistic," and Pierre François Verhulst developed it as a model of population growth in the years between the 1830s and 1840s (Logistic regression—Wikipedia 2020). Later many researchers worked on this function development; the multinomial model called "logit" was introduced by Cox (1966) and Theil (1969) in 1966 and 1969. McFadden (1973) linked the multinomial function logit to the theory of discrete choice, showing that it came from the assumption of independence of irrelevant alternatives as relative preferences. It laid a theoretical foundation for the new logistic regression concept (Cramer 2005). Logistic regression is useful for many applications such as Machine learning, medical, Social sciences. This technique is also useful for engineering, especially for predictingthe success or failure of a system or model. A brief explanation of logistic regression is given below:

Logistic regression predicts the probability in two values only, while a linear regression predicts the values outside the range of (0–1).

The logistic regression calculated using:

1 $$p = \frac{1}{{1 + e^{{ - \left( { + b_{1} x_{1} + b_{2} x_{2} + \cdots + b_{p} x_{p} } \right)}} }}$$

where p = logistic model predicted probability, x = feature or attribute, b_i_ = changes in values of x.

2. Decision Tree Classifier

A decision tree is a supervised learning-based predictive modeling tool created by Quinlan (1986) at the University of Sydney and published in his book Machine Learning. This tool works on the principle of multivariate analysis, which can help predict, explain, describe, and classify the outcome. It splits the dataset based on multiple conditions, thus allowing it to describe beyond one cause cases and helping us describe the condition based on multiple influences. Quinlan (1986) created Iterative Dichotomiser version 3 (ID3) algorithms used to generate decision trees. He then expanded his research based on ID3 and created C4.5, an improved version of ID3. Quinlan is selling a feature-rich and improved version over C4.5, which Quinlan is selling, is C5.0 under GPL. First, a decision tree is generated from the root following the top-down approach that involves data partitioning. Then, entropy and Gini index are calculated using the formula given below.

2 $$Gini = 1 - \mathop \sum \limits_{i = 1}^{C} \left( {p_{i} } \right)^{2}$$

3 $$Entropy = \mathop \sum \limits_{i = 1}^{C} - p_{i} *log_{2} \left( {p_{i} } \right)$$

where P_i_ = probability of ith item. C = class labels. i = iteration.

Many algorithms are used for the generation of decision trees.

I. Classification and regression tree (CART)
II. ID3
III. CHAID
IV. ID4.5

3. Support Vector Machine Classifier

Support Vector Machine (Master Machine Learning Algorithms 2020) Classifier is a supervised learning helpful tool for regression and classification. The central theme of the working of SVM is that it's a binary classification algorithm that separates the data points to find a hyperplane in case of many possible inputs. It handles the outliers efficiently and works well in the case of high-dimensional spaces. It uses decision functions, also known as support vectors, to perform classification. Atleast 4 types of kernels are used for classification SVC with linear kernel, Linear SVC, SVC with RBF kernel, and SVC with the polynomial kernel. The mathematical representation of the classifier algorithm is as under.

4 $$\left[ {\frac{1}{n}\mathop \sum \limits_{i = 1}^{n} {\text{max}}\left( {0,1 - y_{i} \left( {w \cdot x_{i} - b} \right)} \right)} \right] + \lambda \left| {\left| w \right|} \right|^{2}$$

where: w = weight vector, x = input vector, b = bias.

4. K-Nearest Neighbour Classifier

K-Nearest Neighbor (Master Machine Learning Algorithms 2020) is the most simple classifier algorithm, yet it can give a reasonable amount of accuracy in predictions through classification when applied. It is a supervised learning classifier that works well for classification and regression. It is non-parametric and can classify unknown objects based on the K value. The no of training examples is the value of K and plays a vital role in the algorithm because the nearest neighbors of this value are chosen based on a distance function. The distance functions used for this are Minkowski, Manhattan, Hamming, and Euclidian. It is a slow classifier because it needs that full training dataset is to be loaded for classification. All three Mathematical representations of the algorithm are as under.

567where: x_i_ = attribute variable, y_i_ = attribute variable, k = nearest neighbors.

5. Gaussian Naïve Bayes Classifier

Gaussian Naïve Bayes (Master Machine Learning Algorithms 2020) is a supervised learning algorithm used for predictive modeling that works based on the Bayes theorem. Ideally, it is used in the case of continuous data using binning, and it uses the maximum likelihood method. It is suitable for both the cases when binary classification is needed and when multiclass classification is required. It uses a probabilistic approach which involves the probabilistic calculation of the dataset and test data for classification and prediction. The mathematical representation of the algorithm is as below.

8 $$P\left( {c|x} \right) = \frac{{P\left( {x|c} \right)P\left( c \right)}}{P\left( x \right)}$$

9 $$P\left( {c|X} \right) = P\left( {x_{1} |c} \right) \times P\left( {x_{2} |c} \right) \times \cdots \times P(x_{n} |c) \times P\left( c \right)$$

where: P = probability, x = feature, c = class.

6. Linear Discriminant Analysis Classifier

Linear Discriminant Analysis, also known as LDA, is a dimensionality reduction algorithm. It is a supervised classification technique. It was developed by Ronald A. Fisher in the year 1936 and earlier named Fisher's Discriminant Analysis (Fletcher and Reeves 1954). C. R. Rao later generalized a multiclass version called Multiple Discriminant Analysis (Rao 2011). All of these are called Linear Discriminant Analyses. It is often used for performing pattern classification while doing preprocessing of data. Its primary objective is specialization from a generalization approach that eventually reduces dimensional cost and resources. It is generally used in image processing and predictive marketing analysis. It can be mathematically expressed as:

10 $$P\left( {Y = x|X = x} \right) = \left[ {\left( {Pk*fk\left( x \right)} \right)} \right]/[(sum\left( {P|*f\left( x \right))} \right]$$

where p = probability, Y = Class vector, X = feature vector, f = linear score function.

#### Ensemble based classifiers

**Fig. 2:**
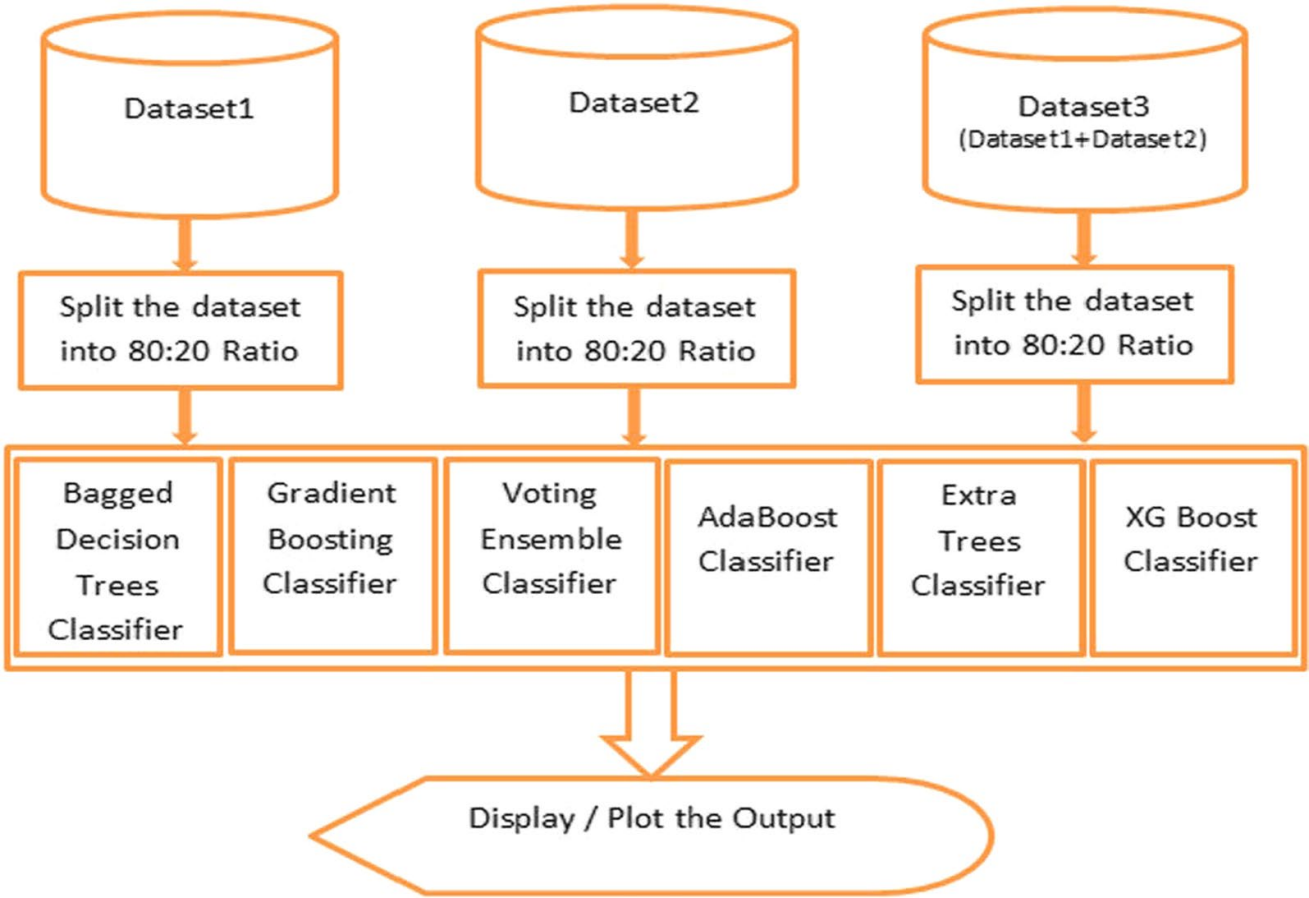
Ensemble Classifier Process chart without cross-validation with datasets 1, 2, and dataset 3

In the next stage, ensemble-based machine learning classifiers are used. At first, the Bagging Classifier is used. Second, the Adaboost Classifier is used. Next, the Gradient Boosting Classifier is used, then the Voting Ensemble Classifier has been used. Finally, the Extra Trees Classifier has been used, followed by XGBoost Classifier. We have incorporated tenfold cross-validation for training testing of the models and performing the analysis on the dataset. The process chart of these ensemble-based classifiers without cross-validation is shown below in Fig. 2.All the individual ensemble-based classifiers are described next to the process charts with and without validation.

### Ensemble classifiers used

1. Bagged Decision Trees (Bagging) Classifier

Bagging classifier (Kleinberg 2000) is named so because it creates an ensemble of decision trees, and it is used for classification or regression. It is also known as bootstrap aggregation. Input data replica is drawn independently in every tree to grow. To handle regression problems, it supports mean and quantile regression.

2. Adaboost Classifier

Adaboost algorithm (Breiman 2001) is named so from the Adaptive Boosting, and It is considered the first binary classification algorithm; there are other approaches built by using boosting features of this algorithm, such as Gradient boosting machines algorithm. Adaboost is implemented using short decision trees. After creating the first tree, its performance is used in each instance of training is used, to weigh how much attention will be given to the next tree, so the most weight is assigned to the tree that contains data that requires more effort for training and prediction, it is easy to predict using less weighted trees.

3. Gradient Boosting Classifier

Gradient boosting algorithm (Friedman 1997) is based on the boosting method used to perform regression, classification, and ranking. It uses an ensemble of multiple weak learners that learn from weak learners iteratively to build a robust model by exploiting the best from all of these weak learner algorithms. The ensemble (group) of weaker classifier algorithms collected together is formed to implement the ensemble classifier algorithms. The collected vulnerable learners are modified to make better classifications.

4. Voting Ensemble Classifier

As per all the ensemble concept-based algorithms that involve the combination of multiple base learning classifier algorithms to improve predictions made by these models and further to combine the individual predictions made by these base classifier models to obtain the final predictions, the voting ensemble classifier algorithm (Koray et al. 2019) and stacking does not require base classifiers to be homogeneous, they can have a mix of different types of algorithms to get the prediction performed and eventually combining the results produced by these classifier algorithms to take the best out of them. The voting ensemble classifier performs two kinds of voting: hard voting and soft voting. In the former approach, the majority vote is the key to be considered, i.e., the frequency of predictions among the models is chosen. In the latter method, the average of the predictions performed by different models is considered. The average is taken from the probability predicted by the base models, constituting the voting classifier.

5. Extra Trees Classifier

The extra trees classifier algorithm (Srinivasa 2019) extends or modifies the bagged decision trees classifier. In this approach, the trees are created from the dataset samples used for training. The extra trees algorithm is very similar to the random forests algorithms in which the optimal feature or split combination is taken to split the dataset as both of these algorithms use the basic functionality of decision trees that split the dataset recursively to construct the tree. A random number or value is used to split the dataset to build the decision tree in the extra trees algorithm. As numerous decision trees are used in random forest classifiers and the extra trees are considered an extension, it is sometimes referred to as extreme random forests.

6. XGBoost Classifier

XGBoost Algorithm (Ali 2017) is a decision Tree-based gradient boosting classifier algorithm. It involves a set of weak learners combined for making predictions. It is a sequential ensemble that improves or corrects the mistakes committed by previous models, where the models are constructed by making corrections to the previous model's misclassification.

### Ensemble classifiers 2nd stage

The classifiers mentioned above are again used on the second dataset, and in this stage, we also used tenfold cross-validation while classifying using these classifiers.

#### Classifying on the merged dataset

**Fig. 3:**
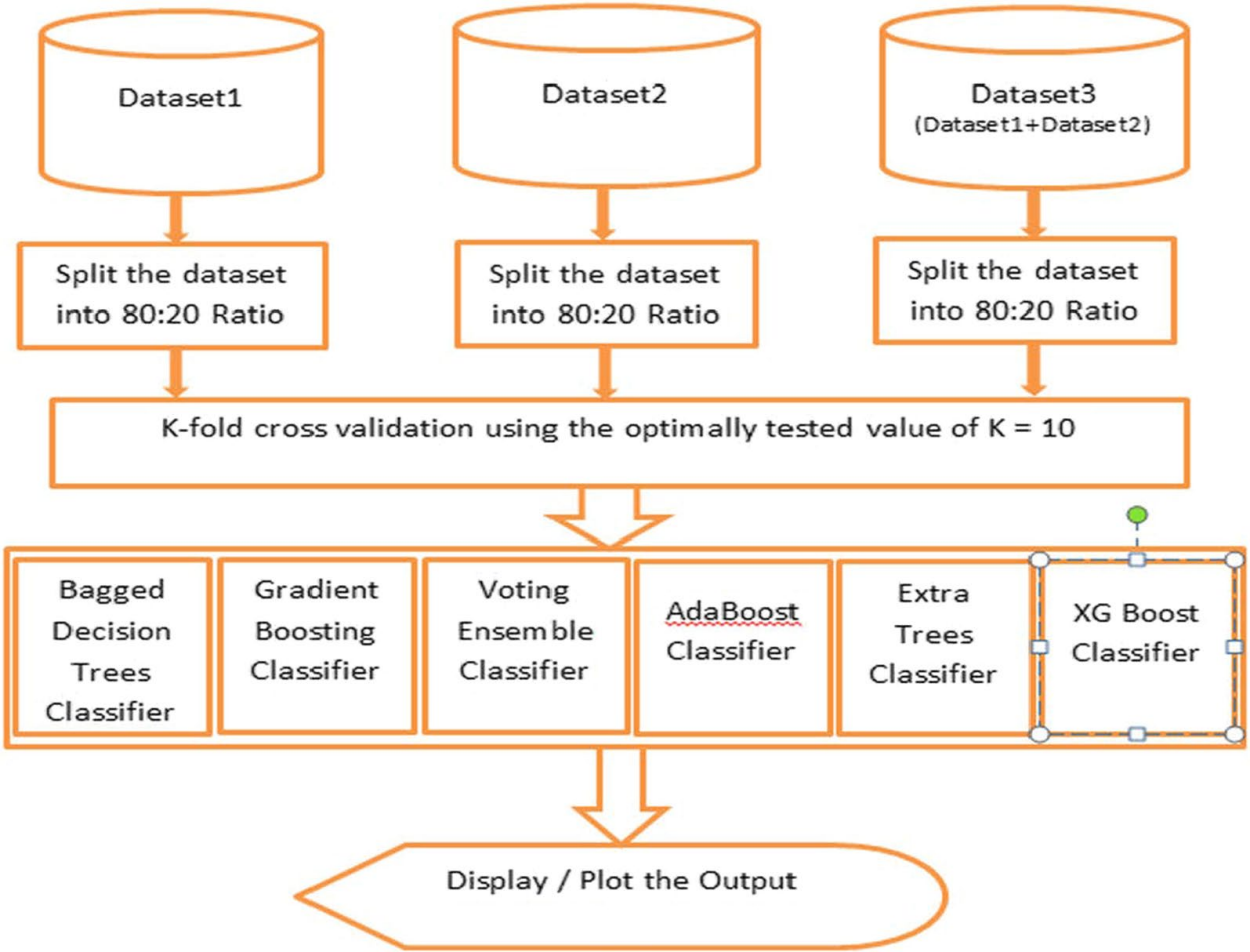
Ensemble Classifier Process chart with datasets 1, 2 and 3 with k-fold cross-validation

The datasets, i.e., first and second datasets containing 2056 and 11,055 instances, have been merged, and the resultant dataset contains 13,511 instances. All the ensemble classifiers have been applied to this merged dataset. Since the dataset contains more instances, it took more time to classify, and the results are degraded only by a bit of difference, which is discussed in the "Results and discussion" section. The process chart with cross-validation for this process is given below in Fig. 3.

### K-fold cross-validation

It is a technique by which the holdout method can be improved. It alters how we pick our dataset for training and testing purposes; instead, it divides the dataset into k number of subsets and repeats the holdout method k number of times. The steps for K-fold cross-validation are.

1. It splits our dataset into k number of subsets called folds.
2. For each fold created from our dataset, builds a model on k–1 folds that are created tests the model to check the model's effectiveness for kth fold afterward.
3. Repeats this process until each k-fold is tested by making them independent test sets.
4. The results are recorded, and the average of the predicted accuracy is then taken out and presented as the test metric for the model currently being tested or implemented.

We have incorporated the k-fold cross-validation for each of the ensemble-based classifiers. We have tested different values of K and picked the most optimal value of K = 10 in our experiment.

## Results and discussion

The first dataset used in the present paper is obtained from the UCI Machine Learning Repository, which contains 2456 instances of website URL data having 31 distinct attributes. The second dataset is obtained from the Kaggle.com repository that contains 11,055 instances and the same 31 attributes in the dataset. In both the datasets, the 30 attributes contain URL features, and the remaining one (1) attribute out of the 31 total attributes, that is labeled as a result contains the values that denote − 1 as (Phishing website), oneas (non-phishing website) and 0 as (Suspicious website) based on URL features. Although the dataset is already preprocessed, we have performed standardization of data to ensure the correct and smooth processing while performing classification and prediction on the above datasets. A detailed description of the dataset is given below in Table 1.

**Table 1:** Description of the attributes in dataset1 and dataset 2

S. no	Column (attribute) name	Column description	Data type	Possible values
1	"having_ip"	Check if IP Address found in URL	Categorical	{1,0}
2	"url_length"	Check if URL length longer than normal	Categorical	{1,0, − 1}
3	"shortining_service"	Check if Shortening Service used	Categorical	{0,1}
4	"having_At_Symbol"	Check if there is @ Symbol in URL	Categorical	{0,1}
5	"double_slash_redirect"	Check if double slashes redirect used	Categorical	{1,0}
6	"prefix_suffix"	Check if Abnormal Prefix and Suffix	Categorical	{− 1,0,1}
7	"having_sub_domain"	Check if Sub Domain in URL	Categorical	{− 1,0,1}
8	"sslfinal_state"	Check if SSLfinal State in URL	Categorical	{− 1,1,0}
9	"Domain_registration_length"	Check if Registration Timeline of domain	Categorical	{0,1, − 1}
10	"favicon"	Check if Favicon used	Categorical	{0,1}
11	"port"	Check enabled ports	Categorical	{0,1}
12	"https_token"	Check if HTTPS token in URL	Categorical	{1,0}
13	"Request_url"	Check if Another URL requested	Categorical	{1, − 1}
14	"url_of_anchor"	Check if URL of Anchor used	Categorical	{− 1,0,1}
15	"links_in_tags"	Check if Links in tags	Categorical	{1, − 1,0}
16	"SFH"	Check if Use of Server Form Handler	Categorical	{− 1,1}
17	"submitting_to_email"	Check if Send data to an email	Categorical	{1,0}
18	"abnormal_url"	Check if Abnormality in URL	Categorical	{1,0}
19	"redirect"	Check if Redirection used	Categorical	{0,1}
20	"on_mouseover"	Check if on_mouseover event used	Categorical	{0,1}
21	"right_click"	Check If RightClick disabled	Categorical	{0,1}
22	"popUpWindow"	Check for calls for PopUp Window	Categorical	{0,1}
23	"iframe"	Check if Iframe is ambiguous	Categorical	{0,1}
24	"age_of_domain"	Check if Too short age of domain	Categorical	{− 1,0,1}
25	"dns_record"	Check if DNS Record is not present	Categorical	{1,0}
26	"web_traffic"	Check if less web traffic present	Categorical	{− 1,0,1}
27	"page_rank"	Check if Inappropriate Page_Rank	Categorical	{− 1,0,1}
28	"google_index"	Check If not in Google Index	Categorical	{0,1}
29	"Links_pointing_to_page"	Check if Links pointing to external page	Categorical	{1,0, − 1}
30	"statistical_report"	Check if Inappropriate Statistical report	Categorical	{1,0}
31	"Result"	Contains Result	Categorical	{1, − 1}

The study was conducted with all 30 features, considering all features are essential. The study tests the impact of all attributes on the performance of the classifiers used to predict phishing websites.

The experiments performed or conducted in this paper highlight the best outcome. The best accuracy has been achieved using Extra Trees and XGBoost classifier algorithms with 99.18% accuracy on two datasets selected for these experiments from UCI Machine Learning Repository: Phishing Websites Data Set (UCI Machine Learning and Repository: Phishing Websites Data Set 2020) and Kaggle.com (Phishing website dataset | Kaggle 2020). The datasets are preprocessed, normalized datasets that contain 30 distinct URL features, plus 1 result attribute, which contains numerical values derived from the data and 2456 instances in the first and 11,055 instances in the second dataset of website URL data. These values are used to determine whether a website is phishing, non-phishing, or suspicious.

**Fig. 4:**
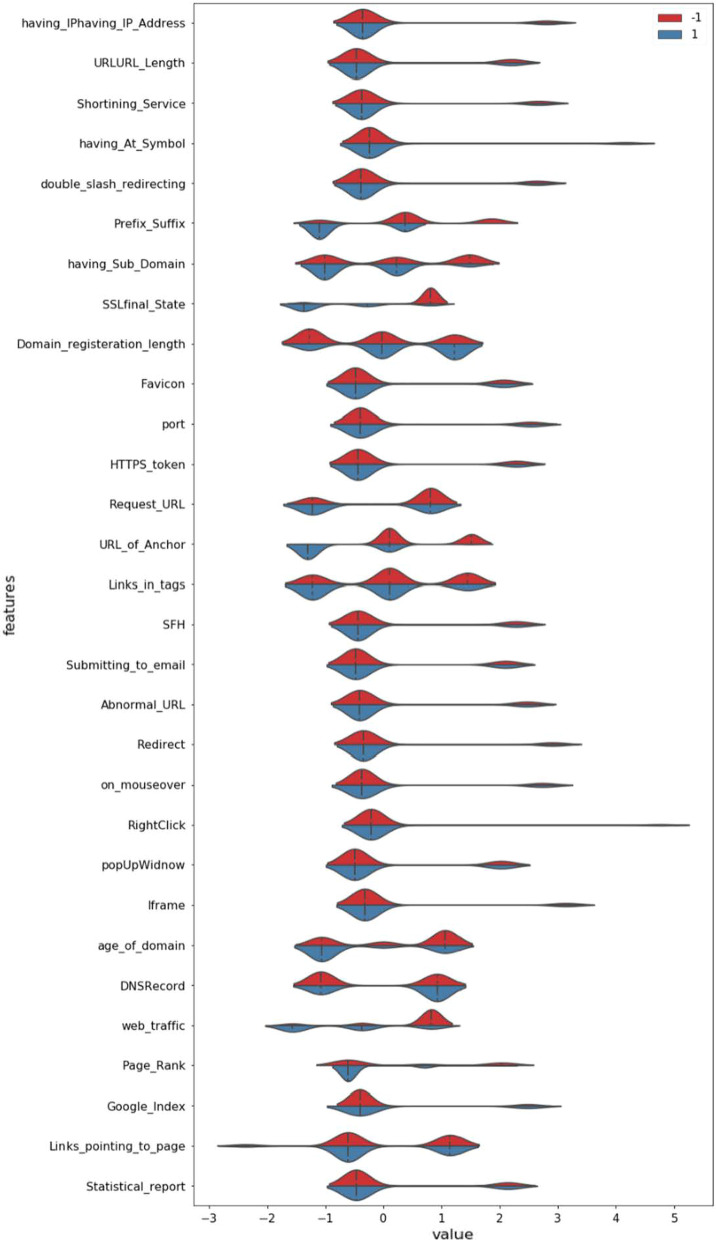
Violin Plot showing Feature distribution in dataset 1

**Fig. 5:**
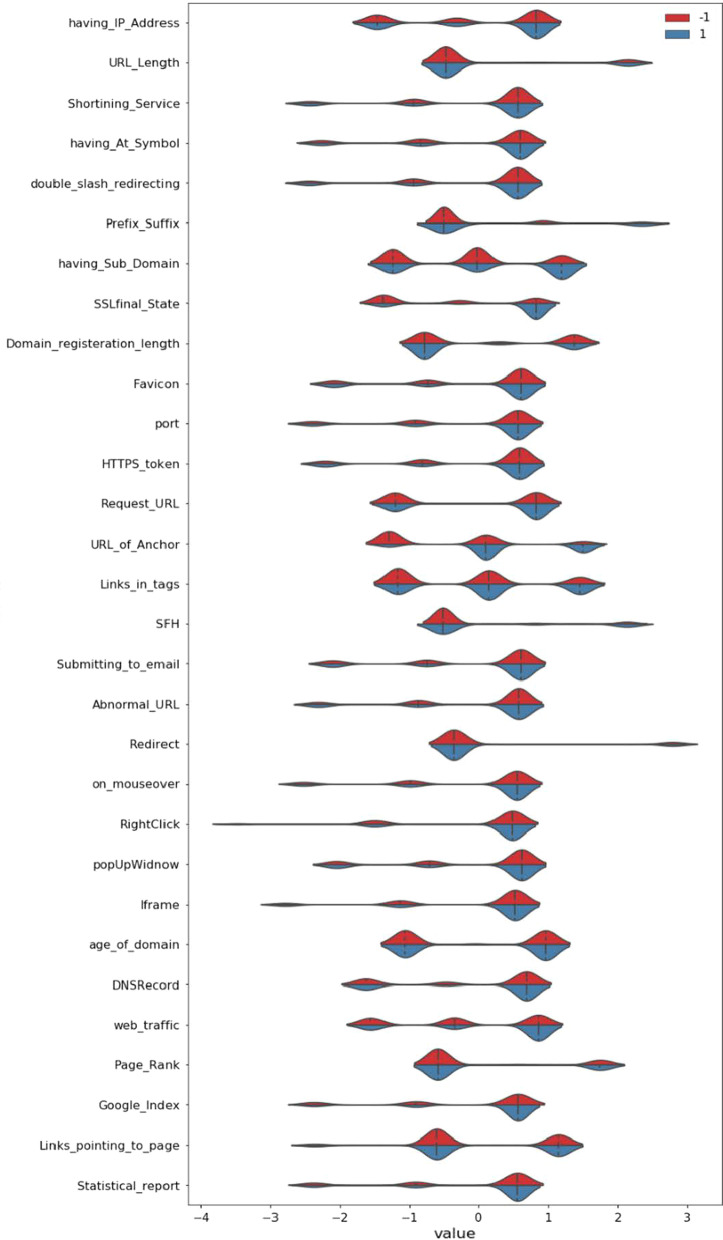
Violin Plot showing Feature distribution in the merged dataset

The exploratory data analysis (EDA) has been performed on the first dataset and the merged dataset using python code and, since the dataset is already in good shape, despite the standardization of data is performed. After the process, all the feature descriptions are given in the graphical representation through the violin plot below in Figs. 4 and 5.

To show the summary statistics of the features in the dataset presented in Figs. 4 and 5 using violin plot. This violin plot represents the distribution of classes (phishing or non-phishing for respective features with color scheme). Figure 4 represents UCI dataset distribution with data size 2456, whereas Fig. 5 represents the merged dataset of UCI and kaggle, both with data size 13,511. Figures 4 and 5 collectively interprets that the merged file has a more significant data distribution than the UCI dataset. The entire study was conducted with a selected algorithm applied over UCI, kaggle, and merged datasets to find the most appropriate predicting algorithm for phishing.

In the present paper, the machine learning classifiers are used in two stages, at first, the logistic regression classifier is used, second, the Gaussian Naïve Bayes classifier, next, the Decision Tree Classifier is used. After that, the Support Vector Machine classifier and the K-Nearest Neighbor classifier are followed by the Linear Discriminant Analysis classifier. Finally, all these classifiers are described in the "Methodology" section above.

A detailed description of the results obtained from base classifiers are given in Table 2 below:

**Table 2:** Classification report with accuracy from experiment 1: base classifiers

Classifier used	Confusion matrix	Non-phishing (− 1) or phishing (1)	Precision	Recall	F1-score	Support	Accuracy achieved (%)
Logistic regression	[[269 11] [10 202]]	− 1 1	0.96 0.95	0.96 0.95	0.96 0.95	280 212	93.28
Linear discriminant analysis	[[268 12] [14 198]]	− 1 1	0.95 0.94	0.96 0.93	0.95 0.94	280 212	92.87
K-nearest neighbor	[[266 14] [8 204]]	− 1 1	0.97 0.94	0.95 0.96	0.96 0.95	280 212	93.43
Decision tree	[[274 6] [3 209]]	− 1 1	0.99 0.97	0.98 0.99	0.98 0.98	280 212	95.92
Gaussian Naïve Bayes	[[255 25] [10 202]]	− 1 1	0.96 0.89	0.91 0.95	0.94 0.92	280 212	91.44
Support vector machines	[[270 10] [11 201]]	− 1 1	0.96 0.95	0.96 0.95	0.96 0.95	280 212	94.80

*Explanation* The data obtained per the metadata discussed in Table 1 were processed with multiple algorithms. Table 2 has 8 columns. The first column is the classifier used for representing the respective classification algorithm name. The second column represents the confusion matrix for the respective classification algorithm. The third column represents the phishing or non-phishing category for the respective algorithm and respective row of the confusion matrix. Similarly, Precision, recall, f1 score support, and accuracy achieved in subsequent columns represent respective values for specific algorithms.

The first algorithm is logistic regression which produces a precision value of 0.96 for non-phishing and 0.95 for phishing; recall value 0.96 for non-phishing and 0.95 for phishing; F1 score value 0.96 for non-phishing and 0.95 for phishing; total accuracy received is 93.28 percent.

The second algorithm is Linear Discriminant Analysis which produces a precision value of 0.95 for non-phishing and 0.94 for phishing; recall value 0.96 for non-phishing and 0.93 for phishing; F1 score value 0.95 for non-phishing 0.94 for phishing; total accuracy received is 92.87 percent.

The Third algorithm is K-Nearest Neighbor, which produces a precision value of 0.97 for non-phishing and 0.94 for phishing; recall value 0.95 for non-phishing and 0.96 for phishing; F1 score value 0.96 for non-phishing and 0.95 for phishing; total accuracy received is 93.43 percent.

The fourth algorithm is Decision Tree which produces a precision value of 0.99 for non-phishing and 0.97 for phishing; recall value 0.98 for non-phishing and 0.99 for phishing; F1 score value 0.98 for non-phishing and 0.98 for phishing; total accuracy received is 95.92 percent.

The fifth algorithm is Gaussian Naïve Bayes which produces a precision value of 0.96 for non-phishing and 0.89 for phishing; recall value 0.91 for non-phishing and 0.95 for phishing; F1 score value 0.94for non-phishing and 0.92 for phishing; total accuracy received is 91.44 percent.

The Sixth algorithm is Support Vector Machines which produces a precision value of 0.96 for non-phishing and 0.95 for phishing; recall value 0.96 for non-phishing and 0.95 for phishing; F1 score value 0.96 for non-phishing 0.95 for phishing; total accuracy received is 94.80 percent.

**Fig. 6:**
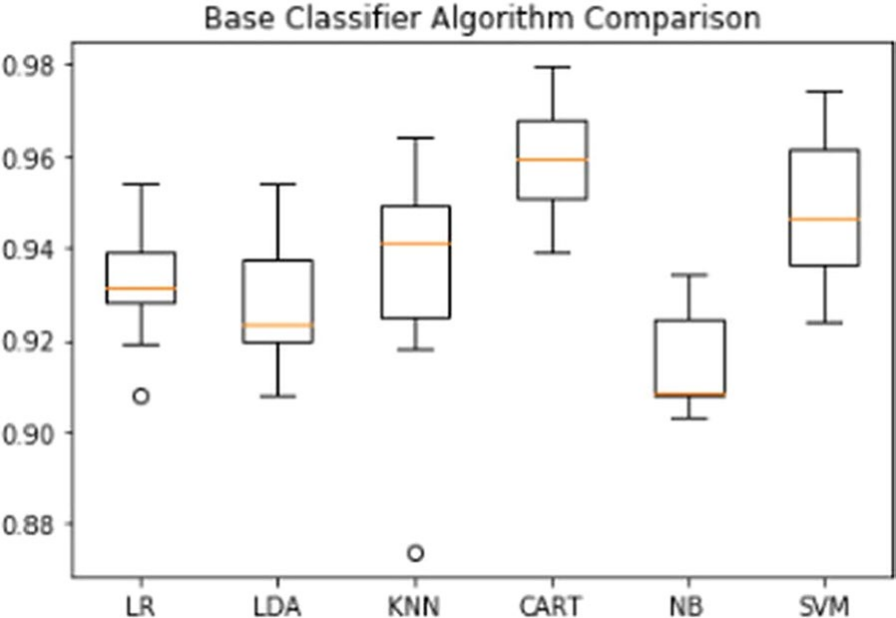
Boxplot representation showing performance of base classifiers

The results are plotted using a boxplot using python code that clearly shows the accuracy scores and the outliers. For example, the boxplot showing the comparison between all the base classifiers used for prediction is shown in the boxplot below in Fig. 6.

### Receiver operating characteristic (ROC) curve analysis

**Fig. 7:**
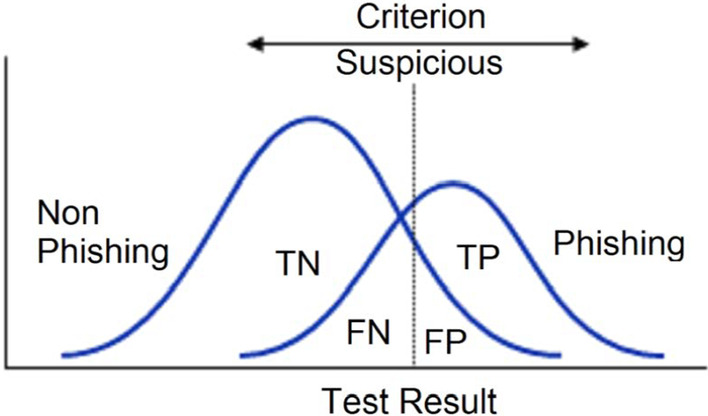
Illustration of overlapping between populations

A ROC curve is a fundamental tool used for diagnostics of test evaluation. It's a common graph used to summarize a classifier's performance overall possible thresholds. It is generated by plotting the true positive rate (TPR) on the y-axis against the false positive rate (FPR) on the x-axis as the threshold is changed for the assignment of observations for a given class. ROC curves can also be used for comparison between 2 tests. ROC may also be used for cost–benefit analysis in the decision-making process. It is used to plot different points of cut-off concerning specificity, also known as false-positive rate. These points represent a pair of sensitivity/specificity pertaining to a specific decision. A ROC curve that passes through very close to the upper left corner denotes a higher level of accuracy and about 100 percent sensitivity and 100 percent specificity. The overlapping may be observed between two sections, i.e., if we consider the populations in the present paper scenario, we seldom find perfect discrimination between these, and instead, we see an overlapping between them. Consider the Fig. 7 below for illustration.

**Fig. 8:**
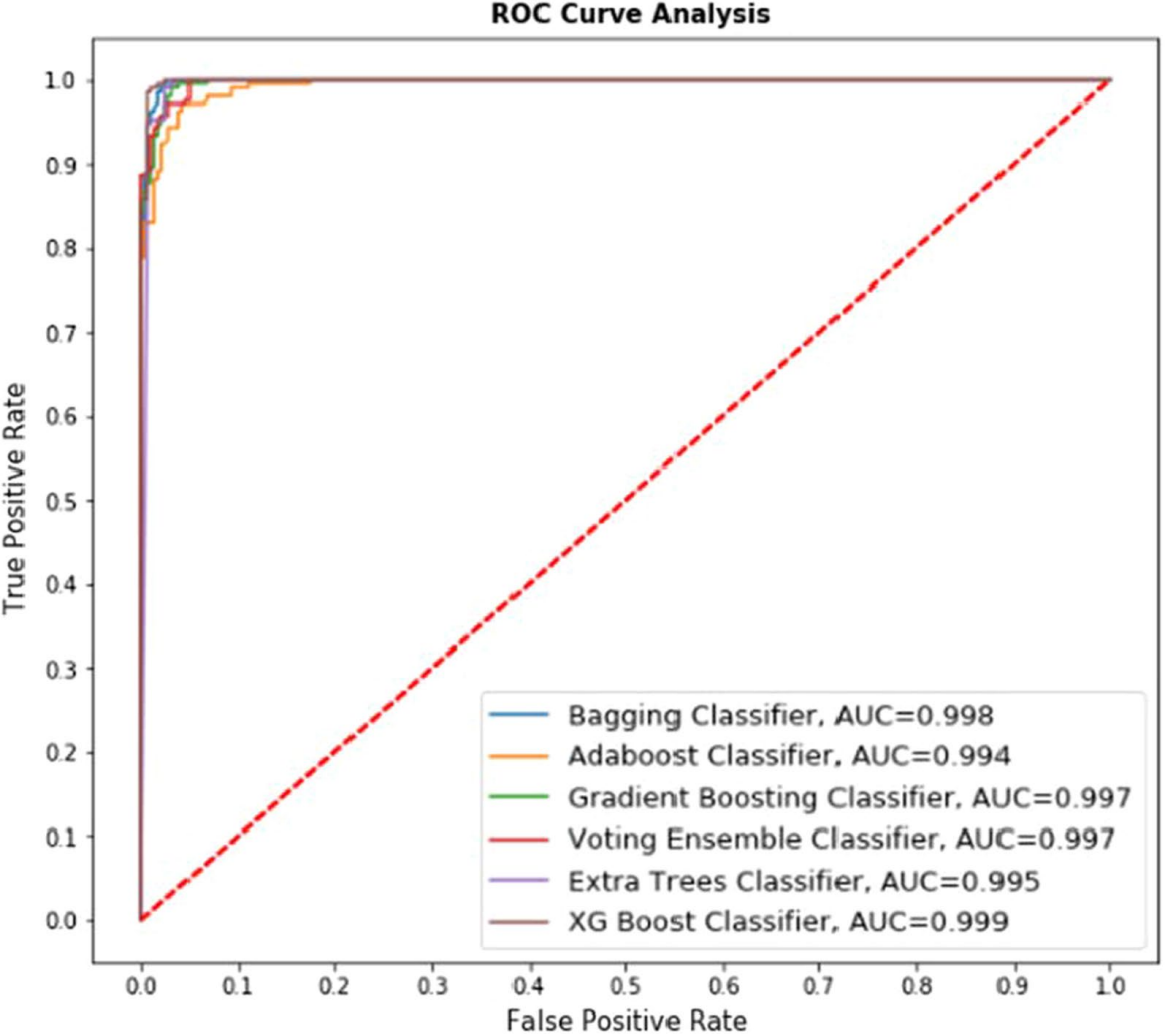
ROC curve for ensemble-based classifiers on UCI dataset

**Fig. 9:**
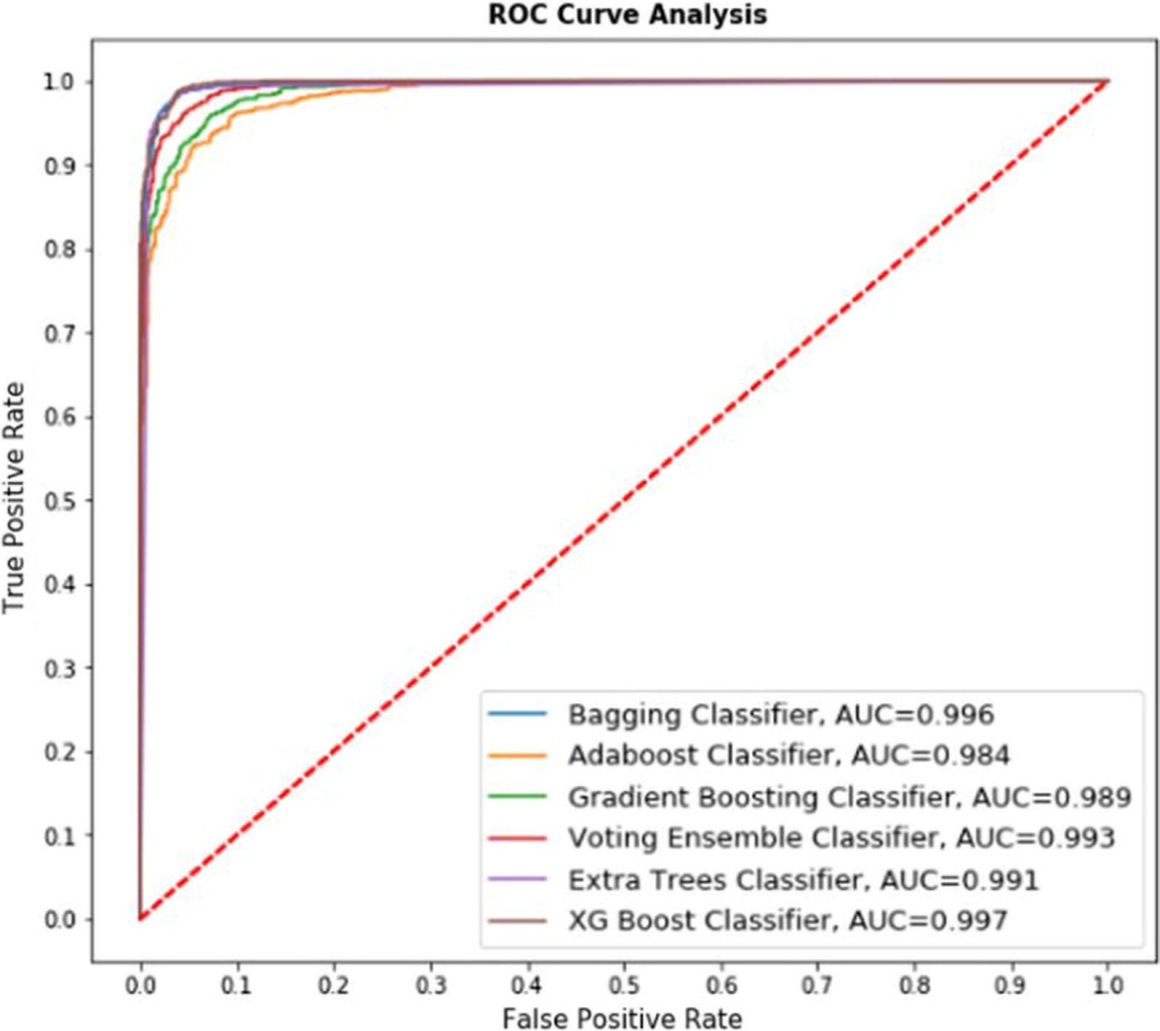
ROC curve for ensemble-based classifiers on Kaggle dataset

**Fig. 10:**
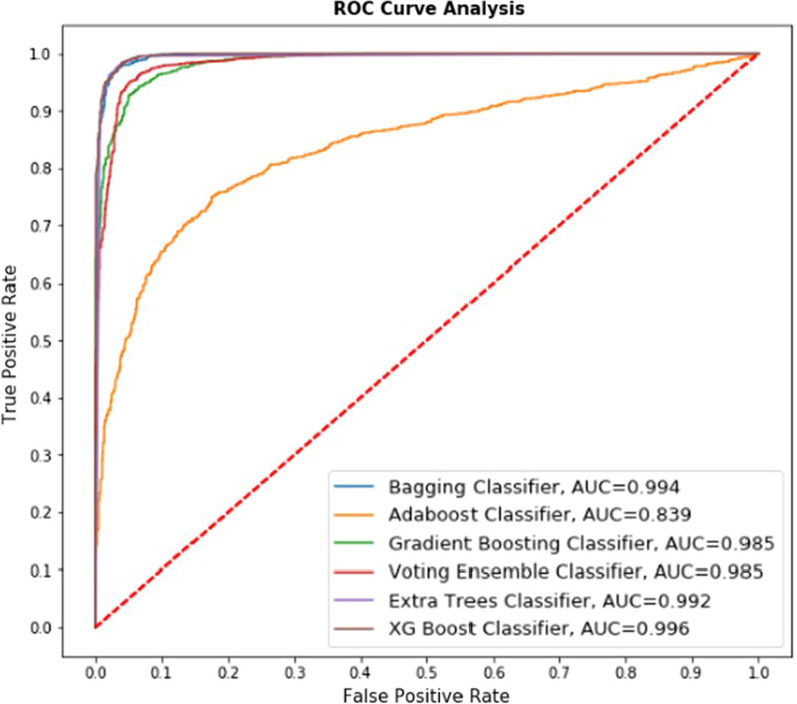
ROC curve for ensemble-based classifiers on the merged dataset without cross-validation

**Fig. 11:**
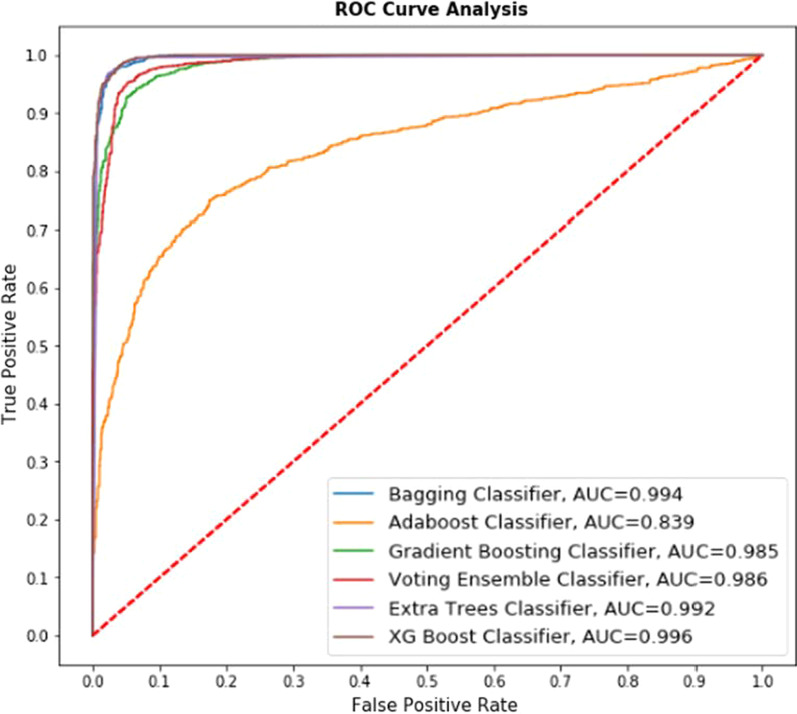
ROC curve for ensemble-based classifiers on the merged dataset with cross-validation

The ROC curves have been plotted for the results obtained by applying all the ensemble-based classifiers on the first, second, and third (merged) datasets described above, given in Figs. 8, 9, 10 and 11 below.

*Explanation* Fig. 8 represents the comparative performance of 6 classifiers (Bagging Classifier, Adaboost Classifier, Gradient Boosting classifier, Voting Ensemble classifier, Extra Trees classifier, XG Boost classifier) on the UCI dataset. The AUC value is presented for the respective classifier in Fig. 8. For example, the lowest AUC value received in Adaboost classifier with value 0.994 whereas highest AUC value received in XG Boost Classifier with value 0.999 and the accuracy obtained using this algorithm is 99.18%, showing the best True Positive rate. So it may be stated that the predicted value is much closer to the value being represented by the ROC curve.

*Explanation* Fig. 9 represents the comparative performance of 6 classifiers (Bagging Classifier, Adaboost Classifier, Gradient Boosting classifier, Voting Ensemble classifier, Extra Trees classifier, XG Boost classifier) on the Kaggle dataset. The AUC value is presented for the respective classifier in Fig. 9. The lowest AUC value received in Adaboost classifier with value 0.984 whereas highest AUC value received in XG Boost Classifier with value 0.997 and the accuracy obtained using this algorithm is 98.37%, showing the best True Positive rate. Comparative study of Figs. 8 and 9 represents that with the increased size of the dataset performance of algorithms declined.

*Explanation* Fig. 10 represents the comparative performance of 6 classifiers (Bagging Classifier, Adaboost Classifier, Gradient Boosting classifier, Voting Ensemble classifier, Extra Trees classifier, XG Boost classifier) on UCI and Kaggle merged dataset. Figure 10 represents performance without cross-validation. The AUC value is presented for the respective classifier in Fig. 10. For example, the lowest AUC value received in Adaboost classifier with value 0.839 whereas highest AUC value received in XG Boost Classifier with value 0.996 and the accuracy obtained using this algorithm is 88.71%, showing the best True Positive rate.

So it may be stated that with the increased size of the dataset, the AUC values shown by ROC curves may sometimes don't give the closest estimation; hence to improve the accuracy, some additional methods may also be incorporated and tested. To do so, cross-validation is incorporated into this merged dataset, and the experiment is conducted.

*Explanation* Fig. 11 represents the comparative performance of 6 classifiers (Bagging Classifier, Adaboost Classifier, Gradient Boosting classifier, Voting Ensemble classifier, Extra Trees classifier, XG Boost classifier) on UCI and Kaggle merged dataset. Figure 11 represents performance with cross-validation. The AUC value is presented for the respective classifier in Fig. 10. For example, the lowest AUC value received in Adaboost classifier with value 0.839 whereas highest AUC value received in XG Boost Classifier with value 0.996 and the accuracy obtained using this algorithm is 98.07%.

Comparative study of Figs. 10 and 11, along with Tables 6 and 7 shows that performance of all selected algorithms increased in the case of using cross-validation. One exciting result obtained for the Extratrees classifier that,although having an AUC value of 0.992, achieved an accuracy of 98.59% is best among all the classifiers.

So it is visible that despite having merged both the datasets and the size of the same has increased, cross-validation should be included in the experiment.

### Confusion matrix

A confusion matrix is a table-like structure in which the performance of a classifier is described or evaluated. This performance description is performed on a dataset for which the true values are already known. Although the terms of the matrix may look confusing, the confusion matrix is usually simple and easy to understand. The terms of the confusion matrix are described below.

*True Positives (TP)* In this case, the prediction is yes (they are Phishing URLs).

*True Negatives (TN)* In this case, the prediction is no (they are not Phishing URLs).

*False Positives (FP)* In this case, the prediction is yes, but it is a false prediction, i.e., they are not the Phishing URLs (It is called a Type I error).

*False Negatives (FN)* In this case, the prediction is no, but they are the phishing URLs (It is called a Type II error).

**Fig. 12:**
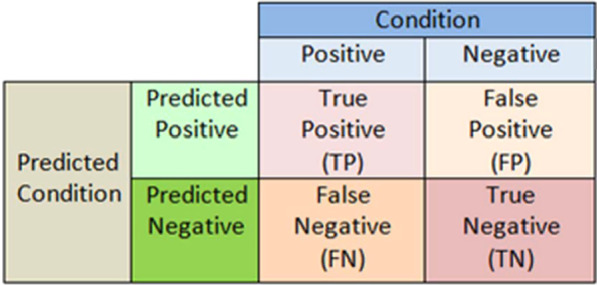
Confusion matrix definition

The confusion matrix values true positive and true negative are obtained from the classifier's expectation, also referred to as prediction, and true and false terms are obtained from external observations. The confusion matrix is formed as in Fig. 12 below.

### Classification report

A classification report is constructed using many constituent variable parameters. These parameters are used to show the values of the parameters used for the calculations of an accuracy score. For example, recall value is known as hit rate, true positive rate (TPR), or sensitivity is obtained using the formula given below in Table. True negative rate (TNR), selectivity also called specificity, is obtained using the formula given below in Table. Positive predictive value (PPV), also known as Precision, is obtained using the formula given below in Table. F1-score or balance f-score, also called traditional f-measure, is the harmonic mean of sensitivity and Precision. Precision is calculated by using the formula as given below in Table 3.

**Table 3:** Confusion matrix metric formula table

Type of metric	Formula	
Accuracy	${\text{ACC}} = \frac{{{\text{tp}} + {\text{tn}}}}{{{\text{tp}} + {\text{fp}} + {\text{tn}} + {\text{fn}}}}$	(11)
Recall	${\text{Recall}} = \frac{{{\text{tp}}}}{{{\text{tp}} + {\text{fn}}}}$	(12)
Precision	${\text{Precision}} = \frac{{{\text{tp}}}}{{{\text{tp}} + {\text{fp}}}}$	(13)
F1-score	${\text{F}} = 2.\frac{{{\text{precision}}.{\text{recall}}}}{{{\text{precision}} + {\text{recall}}}}$	(14)
Specificity	${\text{Specificity}} = \frac{{{\text{tn}}}}{{{\text{tn}} + {\text{fp}}}}$	(15)

### Cohen's Kappa coefficient metric

It is a statistical metric that is useful in measuring how well a classifier has performed and compared to how this classifier would have performed. It is considered to be a more robust measure if it is compared to the simple calculation that represents the scores in percentage. It is calculated mathematically by putting the agreement values of two raters being evaluated. The mathematical representation is given below.

16 $$k \equiv \frac{{P_{0} - P_{e} }}{{1 - P_{e} }} = 1 - \frac{{1 - P_{0} }}{{1 - P_{e} }}$$

where: k = statistic coefficient, p_0_ = relative observed agreement, p_e_ = hypothetical probability of chance agreement.

The performance chart of the ensemble-based classifiers on the first dataset, including classification report, confusion matrix, and kappa score, is given below in Table 4.

**Table 4:** Classification report with accuracy from experiment 1: Ensemble-based classifiers on the first dataset

Classifier Used	Confusion matrix	Kappa score (k)	Phishing (− 1) or non-phishing (1)	Precision	Recall	F1-score	Support	Accuracy achieved (%)
Bagging Classifier	[[274 6] [ 2 210]]	0.967	− 1 1	0.98 0.99	0.99 0.97	0.99 0.98	276 216	98.78
Adaboost classifier	[[272 8] [12 200]]	0.917	− 1 1	0.97 0.94	0.96 0.96	0.96 0.95	284 208	95.91
Gradient boosting classifier	[[2728] [4 208]]	0.950	− 1 1	0.97 0.98	0.99 0.96	0.98 0.97	276 216	97.56
Voting ensemble classifier	[[2728] [6 206]]	0.942	− 1 1	0.97 0.97	0.98 0.96	0.97 0.97	278 214	97.15
Extra trees classifier	[[2728] [2 210]]	0.959	− 1 1	0.97 0.99	0.99 0.96	0.98 0.98	274 218	99.18
XGBoost classifier	[[270 10] [11 201]]	0.971	− 1 1	0.97 1.00	1.00 0.97	0.99 0.98	273 219	99.18

*Explanation* The data obtained per the metadata discussed in Table 4 were processed with multiple algorithms. Table 4 has 7 columns. The first column is the classifier used for representing the respective classification algorithm name. The second column represents the confusion matrix for the respective classification algorithm. The third column represents the phishing or non-phishing category for the respective algorithm and respective row of the confusion matrix. Similarly, Precision, recall, f1 score support, and accuracy achieved in subsequent columns represent respective values for specific algorithms.

The first algorithm is Bagging Classifier which produces a precision value of 0.98 for non-phishing and 0.99 for phishing; recall value 0.99 for non-phishing and 0.97 for phishing; F1 score value 0.99 for non-phishing and 0.98 for phishing; total accuracy received is 98.78 percent.

The second algorithm is Adaboost Classifier Analysis which produces a precision value of 0.97 for non-phishing and 0.94 for phishing; recall value 0.96 for non-phishing and 0.96 for phishing; F1 score value 0.96 for non-phishing and 0.95 for phishing; total accuracy received is 95.91 percent.

The Third algorithm is Gradient Boosting Classifier which produces the precision value of 0.97 for non-phishing and 0.98 for phishing; recall value of 0.99 for non-phishing and 0.96 for phishing; F1 score value 0.98 for non-phishing and 0.97 for phishing; total accuracy received is 97.56 percent.

The fourth algorithm is Voting Ensemble Classifier which produces the precision value of 0.97 for non-phishing and 0.97 for phishing; recall value of 0.98 for non-phishing and 0.96 for phishing; F1 score value 0.97 for non-phishing and 0.97 for phishing; total accuracy received is 97.15 percent.

The fifth algorithm is the Extra Trees Classifier which produces a precision value of 0.97 for non-phishing and 0.99 for phishing; recall value 0.99 for non-phishing and 0.96 for phishing; F1 score value 0.98 for non-phishing 0.98 for phishing; total accuracy received is 99.18 percent.

The Sixth algorithm is XGBoost Classifier which produces a precision value of 0.97 for non-phishing and 1.00 for phishing; recall value 1.00 for non-phishing and 0.97 for phishing; F1 score value 0.99 for non-phishing 0.98 for phishing; total accuracy received is 99.18 percent.

**Fig. 13:**
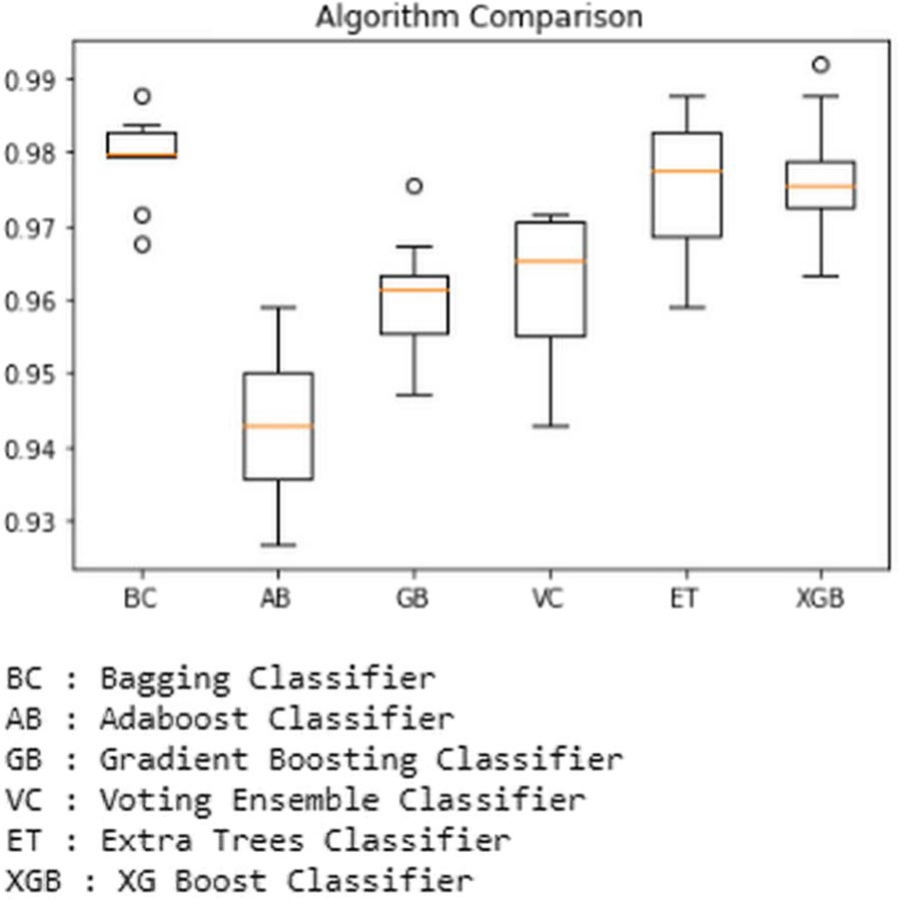
Performance of Ensemble-based classifiers on the first dataset

The results are then plotted using a boxplot using python code that clearly shows the accuracy scores and the outliers. For example, the boxplot showing the comparison between all the ensemble-based classifiers used for prediction is shown in Fig. 13 below.

The performance chart of the ensemble-based classifiers on the Second dataset, including classification report, confusion matrix, and kappa score, is given below in Table 5.

**Table 5:** Classification report with accuracy from experiment 2: Ensemble-based classifiers second dataset

Classifier used	Confusion matrix	Kappa score (k)	Phishing (− 1) or non-phishing (1)	Precision	Recall	F1-score	Support	Accuracy achieved (%)
Bagging classifier	[[954 33] [31 1193]]	0.941	− 1 1	0.97 0.97	0.97 0.97	0.97 0.97	985 1226	98.64
Adaboost classifier	[[898 89] [68 1156]]	0.856	− 1 1	0.91 0.94	0.93 0.93	0.92 0.94	966 1245	94.21
Gradient boosting classifier	[[91671] [531171]]	0.886	− 1 1	0.93 0.96	0.95 0.94	0.94 0.95	969 1242	96.47
Voting ensemble classifier	[[948 39] [631161]]	0.907	− 1 1	0.96 0.95	0.94 0.97	0.95 0.96	1011 1200	97.19
Extra trees classifier	[[954 33] [291195]]	0.943	− 1 1	0.97 0.98	0.97 0.97	0.97 0.97	983 1228	98.73
XGBoost classifier	[[948 39] [17 1207]]	0.949	− 1 1	0.96 0.99	0.98 0.97	0.97 0.98	965 1246	98.37

*Explanation* The data obtained per the metadata discussed in Table 5 were processed with multiple algorithms. Table 5 has 7 columns. The first column is the classifier used for representing the respective classification algorithm name. The second column represents the confusion matrix for the respective classification algorithm. The third column represents the phishing or non-phishing category for the respective algorithm and the respective row of the confusion matrix. Similarly, Precision, recall, f1 score support, and accuracy achieved in subsequent columns represent respective values for specific algorithms.

The first algorithm is Bagging Classifier which produces the precision value of 0.97 for non-phishing and 0.97 for phishing; recall value of 0.97 for non-phishing and 0.97 for phishing; F1 score value 0.97 for non-phishing 0.97 for phishing; total accuracy received is 98.64 percent.

The second algorithm is Adaboost Classifier Analysis which produces a precision value of 0.91 for non-phishing and 0.94 for phishing; recall value 0.93 for non-phishing and 0.93 for phishing; F1 score value 0.92 for non-phishing and 0.94 for phishing; total accuracy received is 94.21 percent.

The Third algorithm is Gradient Boosting Classifier which produces a precision value of 0.93 for non-phishing and 0.96 for phishing; recall value 0.95 for non-phishing and 0.94 for phishing; F1 score value 0.94 for non-phishing and 0.95 for phishing; total accuracy received is 96.47 percent.

The fourth algorithm is Voting Ensemble Classifier which produces a precision value of 0.96 for non-phishing and 0.95 for phishing; recall value 0.94 for non-phishing and 0.97 for phishing; F1 score value 0.95 for non-phishing and 0.96 for phishing; total accuracy received is 97.19 percent.

The fifth algorithm is the Extra Trees Classifier which produces a precision value of 0.97 for non-phishing and 0.98 for phishing; recall value 0.97 for non-phishing and 0.97 for phishing; F1 score value 0.97 for non-phishing 0.97 for phishing; total accuracy received is 98.73 percent.

The Sixth algorithm is XGBoost Classifier which produces a precision value of 0.96 for non-phishing and 0.99 for phishing; recall value 0.98 for non-phishing and 0.97 for phishing; F1 score value 0.97 for non-phishing 0.98 for phishing; total accuracy received is 98.37 percent.

**Fig. 14:**
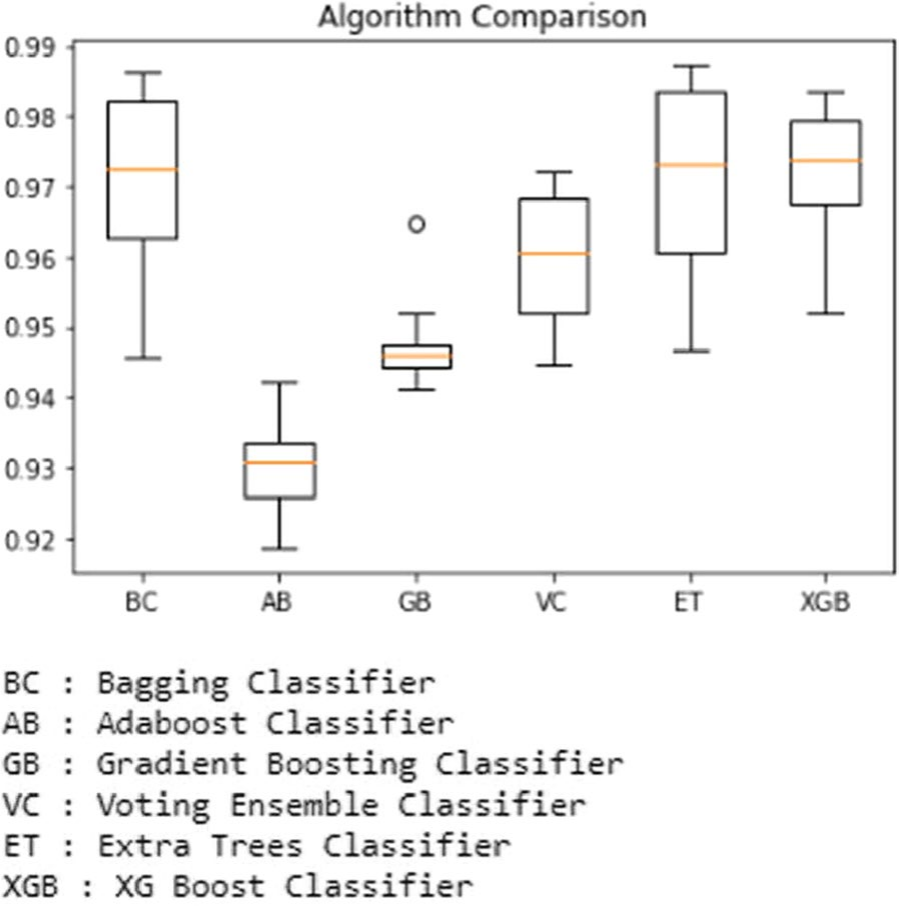
Performance of Ensemble-based classifiers on the second dataset

The results are then plotted using a boxplot using python code that clearly shows the accuracy scores and the outliers. For example, the boxplot showing the comparison between all the ensemble-based classifiers used for prediction is shown in the Fig. 14 below.

The performance charts of the ensemble-based classifiers on the merged dataset, including classification report, confusion matrix, and kappa score with and without the tenfold cross-validation, are given below in Tables 6 and 7.

**Table 6:** Classification report with accuracy from experiment 3: Ensemble-based classifiers on the merged dataset without cross-validation

Classifier used	Confusion matrix	Kappa score (k)	Phishing (− 1) or non-phishing (1)	Precision	Recall	F1-score	Support	Accuracy achieved (%)
Bagging classifier	[[1211 44] [37 1411]]	0.948	− 1 1	0.96 0.97	0.97 0.97	0.97 0.97	1248 1455	89.03
Adaboost classifier	[[891 364] [275 1173]]	0.523	− 1 1	0.71 0.81	0.76 0.76	0.74 0.79	1166 1537	67.41
Gradient boosting classifier	[[1158 97] [74 1374]]	0.873	− 1 1	0.92 0.95	0.94 0.93	0.93 0.94	1232 1471	88.70
Voting ensemble classifier	[[1176 79] [65 1383]]	0.893	− 1 1	0.94 0.96	0.95 0.95	0.94 0.95	1241 1462	84.98
Extra trees classifier	[[1214 41] [35 1413]]	0.943	− 1 1	0.97 0.98	0.97 0.97	0.97 0.97	1249 1454	87.37
XGBoost classifier	[[1207 48] [34 1414]]	0.939	− 1 1	0.96 0.98	0.97 0.97	0.97 0.97	1241 1462	88.71

**Table 7:** Classification report with accuracy from experiment 2: Ensemble-based classifiers on the merged dataset with cross-validation

Classifier used	Confusion matrix	Kappa score (k)	Phishing (− 1) or non-phishing (1)	Precision	Recall	F1-score	Support	Accuracy achieved (%)
Bagging classifier	[[1211 44] [37 1411]]	0.940	− 1 1	0.96 0.97	0.97 0.97	0.97 0.97	1248 1455	98.51
Adaboost classifier	[[891 364] [275 1173]]	0.523	− 1 1	0.71 0.81	0.76 0.76	0.74 0.79	1166 1537	92.52
Gradient boosting classifier	[[1158 97] [74 1374]]	0.873	− 1 1	0.92 0.95	0.94 0.93	0.93 0.94	1232 1471	95.63
Voting ensemble classifier	[[1176 79] [64 1384]]	0.894	− 1 1	0.94 0.96	0.95 0.95	0.94 0.95	1240 1463	96.52
Extra trees classifier	[[1212 43] [35 1413]]	0.942	− 1 1	0.97 0.98	0.97 0.97	0.97 0.97	1247 1456	98.59
XGBoost classifier	[[1207 48] [34 1414]]	0.939	− 1 1	0.96 0.98	0.97 0.97	0.97 0.97	1241 1462	98.07

*Explanation* The data obtained per the metadata discussed in Table 6 were processed with multiple algorithms. Table 6 has 9 columns. The first column is the classifier used for representing the respective classification algorithm name. The second column represents the confusion matrix for the respective classification algorithm. The third column represents the phishing or non-phishing category for the respective algorithm and respective row of the confusion matrix. Similarly, Precision, recall, f1 score support, and accuracy achieved in subsequent columns represent respective values for specific algorithms.

The first algorithm is Bagging Classifier which produces a precision value of 0.96 for non-phishing and 0.97 for phishing; recall value 0.97 for non-phishing and 0.97 for phishing; F1 score value 0.97 for non-phishing and 0.97 for phishing; total accuracy received is 89.03 percent.

The second algorithm is Adaboost Classifier Analysis which produces a precision value of 0.71 for non-phishing and 0.81 for phishing; recall value 0.76 for non-phishing and 0.76 for phishing; F1 score value 0.74 for non-phishing and 0.79 for phishing; total accuracy received is 67.41 percent.

The Third algorithm is Gradient Boosting Classifier which produces a precision value of 0.92 for non-phishing and 0.95 for phishing; recall value 0.94 for non-phishing and 0.93 for phishing; F1 score value 0.93 for non-phishing and 0.94 for phishing; total accuracy received is 88.70 percent.

The fourth algorithm is Voting Ensemble Classifier which produces a precision value of 0.94 for non-phishing and 0.96 for phishing; recall value 0.95 for non-phishing and 0.95 for phishing; F1 score value 0.94 for non-phishing 0.95 for phishing; total accuracy received is 84.98 percent.

The fifth algorithm is the Extra Trees Classifier which produces a precision value of 0.97 for non-phishing and 0.98 for phishing; recall value 0.97 for non-phishing and 0.97 for phishing; F1 score value 0.97 for non-phishing 0.97 for phishing; total accuracy received is 87.37 percent.

The Sixth algorithm is XGBoost Classifier which produces a precision value of 0.96 for non-phishing and 0.98 for phishing; recall value 0.97 for non-phishing and 0.97 for phishing; F1 score value 0.97 for non-phishing 0.97 for phishing; total accuracy received is 88.71 percent.

*Explanation* The data obtained per the metadata discussed in Table 7 were processed with multiple algorithms. Table 7 has 9 columns. The first column is the classifier used for representing the respective classification algorithm name. The second column represents the confusion matrix for the respective classification algorithm. The third column represents the phishing or non-phishing category for the respective algorithm and respective row of the confusion matrix. Similarly, Precision, recall, f1 score support, and accuracy achieved in subsequent columns represent respective values for specific algorithms.

The first algorithm is Bagging Classifier which produces a precision value of 0.96 for non-phishing and 0.97 for phishing; recall value 0.97 for non-phishing and 0.97 for phishing; F1 score value 0.97 for non-phishing and 0.97 for phishing; total accuracy received is 98.51 percent.

The second algorithm is Adaboost Classifier Analysis which produces a precision value of 0.71 for non-phishing and 0.81 for phishing; recall value 0.76 for non-phishing and 0.76 for phishing; F1 score value 0.74 for non-phishing and 0.79 for phishing; total accuracy received is 92.52 percent.

The Third algorithm is Gradient Boosting Classifier which produces a precision value of 0.92 for non-phishing and 0.95 for phishing; recall value 0.94 for non-phishing and 0.93 for phishing; F1 score value 0.93 for non-phishing and 0.94 for phishing; total accuracy received is 95.63 percent.

The fourth algorithm is Voting Ensemble Classifier which produces a precision value of 0.94 for non-phishing and 0.96 for phishing; recall value 0.95 for non-phishing and 0.95 for phishing; F1 score value 0.94 for non-phishing 0.95 for phishing; total accuracy received is 96.52 percent.

The fifth algorithm is the Extra Trees Classifier which produces a precision value of 0.97 for non-phishing and 0.98 for phishing; recall value 0.97 for non-phishing and 0.97 for phishing; F1 score value 0.97 for non-phishing 0.97 for phishing; total accuracy received is 98.59 percent.

The Sixth algorithm is XGBoost Classifier which produces a precision value of 0.96 for non-phishing and 0.98 for phishing; recall value 0.97 for non-phishing and 0.97 for phishing; F1 score value 0.97 for non-phishing 0.97 for phishing; total accuracy received is 98.07 percent.

**Fig. 15:**
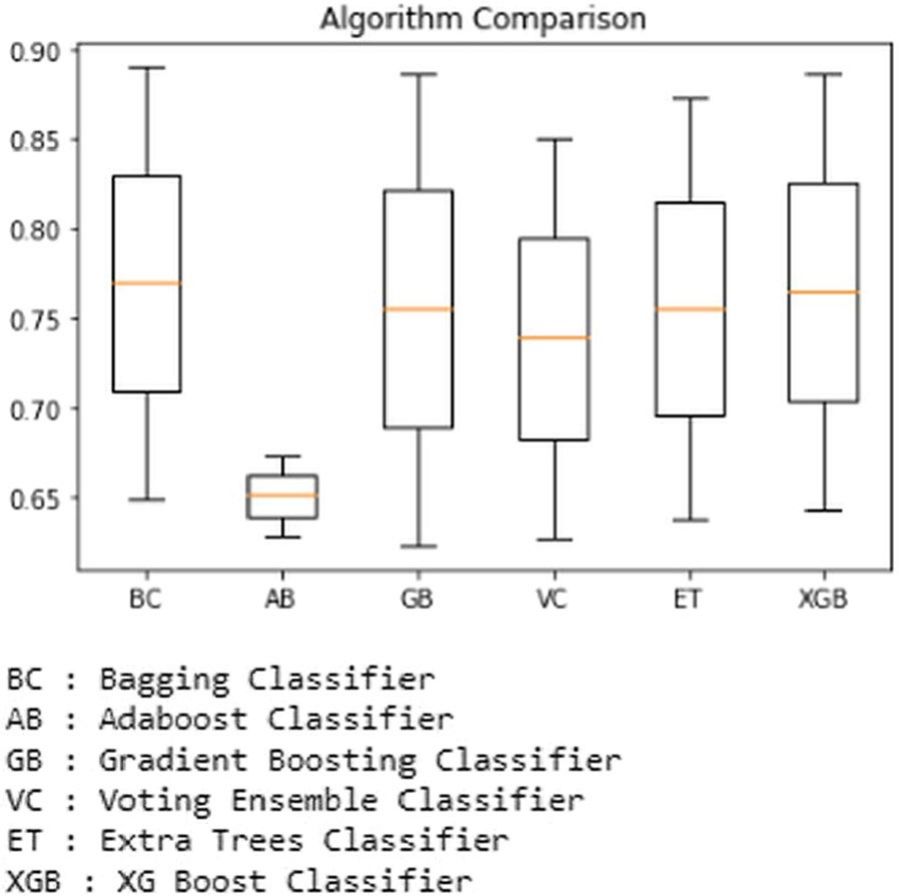
Performance of Ensemble-based classifiers on the merged dataset without k-fold cross-validation

**Fig. 16:**
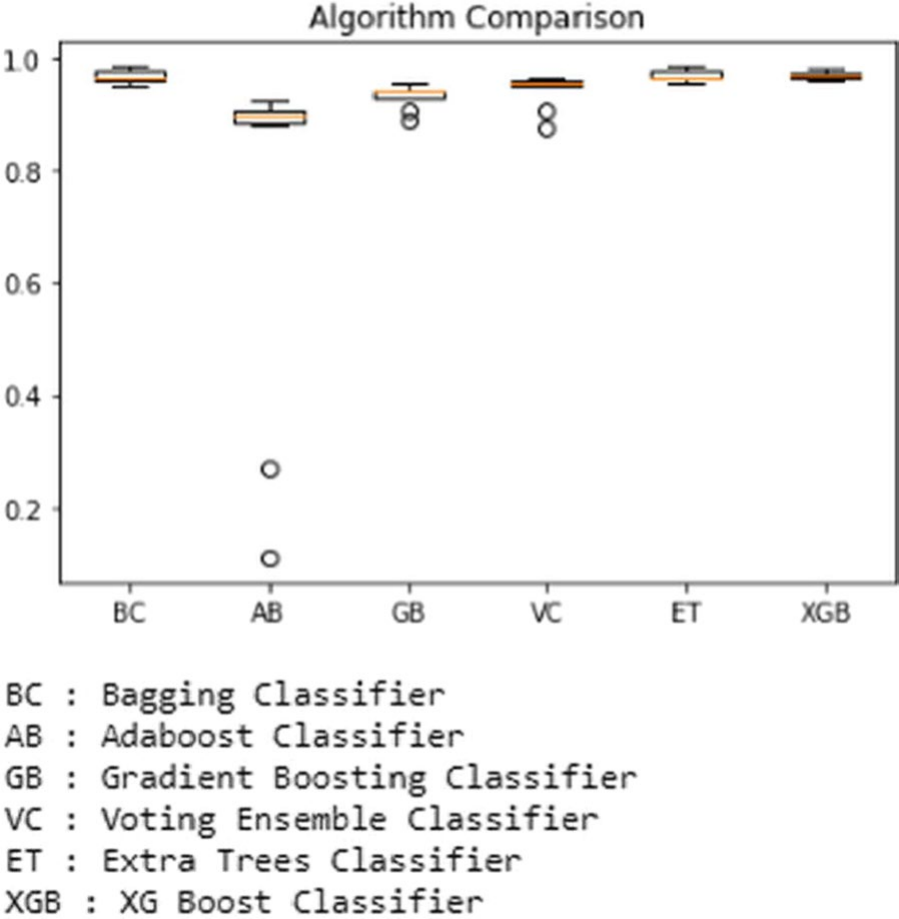
Performance of Ensemble-based classifiers on the merged dataset using k-fold cross-validation

The merged dataset resultsare then plotted using boxplot using python code for both with and without k-fold cross validation that shows the accuracy scores and the outliers. For example, the boxplot comparing all the ensemble-based classifiers used for prediction shows Figs. 15 and 16 below.

In the present experiment, the machine learning classifiers have been implemented using python code. At first, the base classifiers have been used, and the results are; at first, the logistic regression classifier is used, which yielded the accuracy of 93.28 percent, second, the Gaussian Naïve Bayes classifier has given the accuracy of 91.44 percent, next the Decision Tree Classifier is used. It predicted an accuracy of 95.87 percent, while the Support Vector Machine classifier has predicted with an accuracy of 94.80 percent. Then, the K-Nearest Neighbor classifier has been predicted with 93.43 percent accuracy. Finally, the linear Discriminant Analysis classifier has produced 92.87 percent accuracy.

In the next step, we experimented with the first dataset. At first, the Bagging Classifier was used, which yielded an accuracy of 98.78 percent. Second, the Adaboost Classifier is used. It has given accuracy of 95.91 percent. Next, the Gradient Boosting Classifier is used, and it predicted the accuracy of 97.56 percent, while Voting Ensemble Classifier has predicted with an accuracy of 97.15 percent. The Extra Trees Classifier has been predicted with 99.18 percent accuracy. XGBoost Classifier has produced 99.18 percent accuracy. In the next step, we experimented with the second dataset. At first, the Bagging Classifier was used, which yielded an accuracy of 98.64 percent. Second, the Adaboost Classifier is used. It has given accuracy of 94.21 percent. Next, the Gradient Boosting Classifier is used, and it predicted the accuracy of 96.47 percent, while Voting Ensemble Classifier has predicted with an accuracy of 97.19 percent. Finally, the Extra Trees Classifier has been predicted with 98.73 percent accuracy. XGBoost Classifier has produced 98.37 percent accuracy.

In the next step, we experimented on the merged dataset; at first, the Bagging Classifier was used, which yielded an accuracy of 98.51 percent. Second, the Adaboost Classifier is used; it has given accuracy of 92.52 percent. Next, the Gradient Boosting Classifier is used, and it predicted the accuracy of 95.63 percent, while Voting Ensemble Classifier has predicted with an accuracy of 96.52 percent. Next, the Extra Trees Classifier has been predicted with 98.59 percent accuracy. Finally, XGBoost Classifier has produced 98.07 percent accuracy.

In this study, the comparison of results with and without tenfold cross-validation obtained from ensemble-based classifiers is performed, and the results clearly show that ensemble classifiers performed better when using cross-validation while performing classification. As in the cross-validation approach, the datasets are split into as many sections as many folds are selected fork-fold cross-validation as opposed to the regular split into 2 parts when using standard train test split using python. It is proven out of this experimental study that the ensemble-based classification techniques have outperformed the base classifiers. We have changed the dataset in performing this experiment where it has been observed that when at first the number of instances from 2456 to 11,055, the results have been degraded by 0.2 percent to 1.2 percent approximately. But when we merged both the datasets, despite having the same attributes in the resultant dataset and the total number of instances increased to 13,511, the results were affected significantly when the cross-validation was not applied. The maximum degrade observed approximately25 percent in the case of the Adaboost classifier, and the minimum was observed in the case of the Gradient Boosting classifier is off 6.9 percent approximately. Extra Trees and XGBoost Classifiers have given the best accurate prediction output; both have predicted an output score of 99.18 percent with the first dataset and 98.73, 98.37 percent, and 98.07, 98.59 percent on the second datasets, both using cross-validation.

After using different datasets and merging both the datasets, subsequently increasing the number of instances significantly, the prediction accuracy has not been degraded in the case of ensemble classifiers and found to be well ahead of the base classifiers as the minimum accuracy score predicted by the Naïve Bayes classifier at 91.44 percent among the base classifiers and the minimum score predicted by the ensemble-based classifiers is 95.91 percent by AdaBoost classifier. The maximum accuracy score predicted by the Decision Tree classifier is 95.92 among all the base classifiers used, whereas Extra Trees and XGBoost Classifiers have predicted a maximum accuracy score of 99.18 percent. But when classifying on the third, i.e., merged dataset, the best accuracy without k-fold cross-validation was predicted by the XGBoost classifier of 88.71 percent, and with the cross-validation, the best accuracy has been predicted by ExtraTrees classifier of 98.59 percent. So despite having a larger dataset, the ExtraTrees Algorithm performed the best with the cross-validation.

## Conclusion and future scope

In this paper, a study has been performed to address a very important cybersecurity-related aspect that is an important issue faced by almost every level in an organization. This is no more limited to the IT companies or such organizations, which are somehow using network/web technologies to operate their businesses. It is a matter of concern for the entire society because if anybody falls prey to such an attack, it is equally dangerous or harmful for all of them. Phishing is perhaps the oldest kind of computer-based crime that has been the best choice for hackers and cybercriminals worldwide. In this study, we conducted three experiments to detect and predict phishing website URLs using website URL features with the help of machine learning algorithms in 2 stages. We have used two datasets in this study. The first is taken from UCI online machine learning repository having 2456 URL data with 30 distinct features. And second is taken from the kaggle.com repository, having 11,055 URL data with 30 features. In the first stage of the experiment, we tested the accuracy with base classifiers, and later, we also tested for the prediction with ensemble-based machine learning classifiers using the first and second datasets. After this, in the second experiment, we have tested on a merged dataset of the previously mentioned two. In the third experiment, we applied all the ensemble algorithms with and without k-fold cross-validation, and the results are shown in the "Results and discussion" section. The results achieved by this study are very good, and as compared to the previous pieces of literature, we found them to be performing better. Also, we have tested with and without performing cross-validation, so it presented the idea that it is better to perform k-fold cross-validation than predictions made without it.

Since the study in this domain is at the initial stage, we have included all the 30 features for evaluation. However, features will be reduced by selecting the contributing features in various classifiers compared with other dimensionality reduction methods in future studies.

## Data Availability

The dataset is free to use on UCI and Kaggle machine learning repositories.
